# Structural barriers to scientific progress

**DOI:** 10.1107/S2059798320011201

**Published:** 2020-09-22

**Authors:** K. Cowtan

**Affiliations:** a Department of Chemistry, University of York, York YO10 5DD, United Kingdom

**Keywords:** careers, gender, pay, bias

## Abstract

Structural biases, which are intrinsic in the social structures in which we function, play a key role in maintaining boundaries between traditionally privileged and underprivileged groups; however, they are particularly difficult to identify from within those societies. Two instances are highlighted in which the social structures of science appear to have discouraged collaboration, to the disadvantage of software and data users. Possible links are suggested to the strongly hierarchical structure of science and other factors which may in turn also serve to maintain sex and/or gender disparities in participation in the scientific endeavour.

## Abstract 2

A possible connection is discussed between hierarchical and other structures which maintain gender disparities in science and two instances where barriers to collaboration have hindered scientific progress.

The scientific endeavour is a social process which has the potential to benefit the whole of humankind. However, not everyone has equal access to participation in this process, even when comparing people of equal ability; it is well established that scientific enterprise is more accessible to participants who are male (Pell, 1996 ▸; Roper, 2019 ▸; Astegiano *et al.*, 2019 ▸), of privileged ethnicity (Bhopal & Henderson, 2019 ▸) and non-disabled (Inckle, 2018 ▸). This violates our basic notions of fairness and equality, but also impoverishes the scientific enterprise by narrowing the pool of available talent on grounds other than ability, and also by narrowing the range of perspectives present in the pool of scientists thinking about a problem (Powell, 2018 ▸).

Women perform at comparable levels to men in the early stages of their careers; however, they are increasingly poorly represented at later career stages, a difference which remains even when accounting for the effects of career breaks (Blackaby *et al.*, 2005 ▸), suggesting the presence of a systemic bias based on sex and/or gender. Awards (such as the Nobel Prize) and promotions typically disproportionately recognize those at the top of hierarchies for work which has been conducted by a large team of researchers (Lincoln *et al.*, 2012 ▸), a process which reduces the visibility of early-career researchers. Similarly, well established scientists are invited to give keynote talks at conferences (including the first session of the CCP4 Study Weekend), further enhancing their visibility.

Science is perhaps the most effective tool humanity has for distinguishing claims that are objectively true, and its social structures and conventions, including academic institutions, journals, grant panels, consensus and peer review, have to some extent evolved to reduce the impact of subjectivity (Miller, 2013 ▸; Oreskes, 2019 ▸), despite the inevitable errors and cognitive biases of all of the participants (Kahneman, 2011 ▸). However, much of this evolution occurred in an environment when published science was largely produced by financially independent abled white males, and it would therefore be surprising if no systemic biases had been incorporated in these structures.

Systemic and structural biases are particularly hard to recognize when we work exclusively within the system in which the bias is incorporated, and are also hard to demonstrate experimentally because to do so involves deconstructing the system. The early stages of the identification of a systemic bias inevitably involve speculation based on *ad hoc* observations, with the aim of identifying problems for more systematic research.

This letter was inspired by an apparently similar set of circumstances arising in two different fields. When a crystallographer started comparing climate data sets from national science agencies, the differences led rapidly to a number of insights which were not being pursued by more experienced practitioners (Cowtan & Way, 2014 ▸; Hausfather *et al.*, 2017 ▸; Cowtan *et al.*, 2018 ▸). Similarly, when a computer science student started comparing crystallographic model-building software packages (Alharbi *et al.*, 2019 ▸), the resulting insights led to new ways of combining these packages (Alharbi *et al.*, 2020 ▸) and significant improvements to one of them (Bond *et al.*, 2020 ▸). These insights should have been within the reach of practitioners in the fields, but in both cases it took the intervention of a relatively inexperienced outsider to catalyze the progress.

It would be interesting to understand the motivations which led to these events; however, we are not always able to elucidate our own motivations, let alone those of others. One instance of a direct critical comparison across groups is present in Fig. 13 of Hansen *et al.* (2010 ▸): the authors note that a substantial part of the difference between global temperature series arises from differences between the spatial coverage of the data. Hansen *et al.* (2010 ▸) however stop short of highlighting the result, known decades before, that the simple mean of a spatially incomplete field is a biased estimator of the global mean (Kagan, 1979 ▸; Cowtan *et al.*, 2018 ▸). Based on my own experience, I hypothesize that the tension between competition-oriented incentives (for example, the desire to attract users, citations, prestige and funding) and communal benefits (*i.e.* the advancement of science by building on the work of others and having them build on your work) creates a cognitive dissonance which leads to avoidant behaviours.

Fang & Casadevall (2015 ▸) argue that the ‘history of science shows that transformative discoveries often occur in the absence of competition, which only emerges once fields are established and goals are defined’. They highlight cases where competition led to incorrect conclusions, including the triple-stranded model of DNA proposed by Pauling in competition with Watson (Pauling & Corey, 1953 ▸) and the work of Kitasato on the cause of plague (Bibel & Chen, 1976 ▸). High-stress competitive environments, as typified by the current research culture, inhibit creativity (Amabile, 1998 ▸), while even moderately competitive environments may differentially impact the performance of male and female researchers (Amabile, 1996 ▸), with women performing better in more collaborative environments (Baer *et al.*, 2014 ▸).

Hierarchical structures may also play a role both in disincentivizing collaboration and in suppressing creativity, as well as being a possible mechanism by which a structural bias might suppress gender diversity. Male social interaction styles are distinguished by higher levels of dominance signalling and hierarchical behaviours (Maccoby, 1990 ▸; Tannen, 2010 ▸), and testosterone is also implicated in hierarchical behaviours (Eisenegger *et al.*, 2011 ▸; Inoue *et al.*, 2017 ▸), although the connection between these factors is complex and probably involves short-term hormonal influences on behaviour, long-term hormonal influences on brain structure, and social and cultural norms which may or may not derive from physical characteristics (Wood & Eagly, 2012 ▸; Pol *et al.*, 2006 ▸). While there are well established social norms for the communication of ideas within a project hierarchy, the ambiguous relationship between independent hierarchies of scientists working to solve the same problem may hinder collaboration across the borders of a hierarchy (Tsai, 2002 ▸). Established hierarchies can also be resistant to disruptive ideas from early-career researchers and scientists with minority perspectives within those hierarchies (Amabile, 1998 ▸; Neumann, 2007 ▸).

Gender gaps in STEM fields are often attributed to issues of confidence in women (Etzkowitz *et al.*, 2000 ▸; Shen, 2013 ▸), with confidence-boosting measures as a proposed solution (Baker, 2010 ▸; Campbell & Skoog, 2004 ▸). Testosterone levels are known to influence self-confidence (Eisenegger *et al.*, 2011 ▸; Costa *et al.*, 2016 ▸), producing an unequal starting point for men and women, which is in turn reinforced by gendered behavioural norms and unconscious bias (Easterly & Ricard, 2011 ▸). There is a risk therefore that efforts to address gender gaps through confidence-boosting measures alone are in practice teaching women to emulate male behaviours, which contains the implicit assumption that testosterone-dominant behaviours are in some way optimal for scientific investigation (Shansky, 2019 ▸). This male-normative assumption is questionable given that gender-diverse groups produce higher quality science than all-male groups (Campbell *et al.*, 2013 ▸; Hofstra *et al.*, 2020 ▸), and that differences in confidence levels are often reflective of *over*confidence in men (Cho, 2017 ▸). If this is the case, then this kind of approach to addressing the gender gap in science may serve only to conceal the underlying problem rather than addressing it (Black & Islam, 2014 ▸).

In summary, there is evidence that hierarchical behaviours, overly competitive environments and issues relating to confidence all present barriers to the wider participation of women in science. In each case there is substantial evidence of gender differences, with these differences also being correlated with the influence of testosterone in addition to cultural and social factors. In each case there is evidence that in some cases at least these behaviours can be detrimental to the practice of science. Improving participation has often involved training women to perform better in existing social structures by learning behaviours that are more compatible with those structures (Black & Islam, 2014 ▸). I suggest that in some cases this may mitigate some of the symptoms of a deeper structural problem without addressing all of the causes. If these factors are indeed detrimental to the practice of science, then it makes more sense to change the system to both improve the practice of science and reduce the barriers to participation.

Addressing systemic biases of systems of which we are a part is hard, particularly as it is often difficult to see the biases inherent in the system from within, let alone how to address them. I am trying to adopt the following principles and tentatively suggest them as a starting point for other participants.

(i) We must continually listen to the experiences of women in science, to scientists who experience gender in ways distinct from traditional binary norms, and to those who are in the position of having lived with both male-typical and female-typical hormonal profiles, in order to better understand the impact of structural gender biases.
(ii) We should also seek to continually learn from participants in science from other groups that are underrepresented in the scientific community (who are otherwise invisible in this letter), while at the same time learning to recognize how our own preconceptions prevent us from hearing what they are saying.
(iii) We should seek opportunities to disrupt existing hierarchies. Scientific awards and keynote lectures both concentrate credit on established leaders: these should be used as opportunities to draw attention to the work of identified early-career researchers and to highlight the systemic biases which are being reinforced.
(iv) We should recognize that incentive structures created by funding bodies also drive competition and promote hierarchical structures which do not necessarily align with scientific imperatives. We should aim to use funding in ways which mitigate those aspects of the funding scheme which are counterproductive. The increasing priority given by funding bodies to the open-science agenda may help here, although funding bodies may still be constrained in the extent to which they can realize these ambitions.

Adjusting my practice to better align with ethical and scientific goals rather than social and funding norms also leads to some immediate and specific actions. I am offering undergraduate and postgraduate projects to understand how our tools might be incorporated into competing packages in order to improve those packages. I aim to release all future code under Creative Commons licences, and am reviewing past legal agreements to determine the extent to which existing software can be made more reusable. Finally, in the context of computational methods development, I note that it is critical to better understand how traditional teaching methods lead to unequal outcomes when training new generations of scientific programmers (Cooper & Weaver, 2003 ▸), and to develop teaching methods to address this problem.

## References

[bb1] Alharbi, E., Bond, P. S., Calinescu, R. & Cowtan, K. (2019). *Acta Cryst.* D**75**, 1119–1128. 10.1107/S2059798319014918 31793905

[bb2] Alharbi, E., Calinescu, R. & Cowtan, K. (2020). *Acta Cryst.* D**76**, 814–823. 10.1107/S2059798320010542 PMC7466752 32876057

[bb3] Amabile, T. M. (1996). *Creativity in Context*, p. 317. Boulder: Westview Press.

[bb4] Amabile, T. M. (1998). *Harvard Bus. Rev.* **76**(5), 76–87. https://hbr.org/1998/09/how-to-kill-creativity. 10185433

[bb5] Astegiano, J., Sebastián-González, E. & Castanho, C. (2019). *R. Soc. Open Sci.* **6**, 181566. 10.1098/rsos.181566 PMC6599789 31312468

[bb6] Baer, M., Vadera, A. K., Leenders, R. T. & Oldham, G. R. (2014). *Organ. Sci.* **25**, 892–908.

[bb7] Baker, M. (2010). *J. Sociol.* **46**, 317–334.

[bb8] Bhopal, K. & Henderson, H. (2019). *Educ. Rev.*, https://doi.org/10.1080/00131911.2019.1642305.

[bb9] Bibel, D. J. & Chen, T. (1976). *Bacteriol. Rev.* **40**, 633–651. 10.1128/br.40.3.633-651.1976 PMC413974 10879

[bb10] Black, C. & Islam, A. (2014). *The Guardian*. https://www.theguardian.com/higher-education-network/blog/2014/feb/24/women-academia-promotion-cambridge.

[bb11] Blackaby, D., Booth, A. L. & Frank, J. (2005). *Econ. J.* **115**, F81–F107.

[bb12] Bond, P. S., Wilson, K. S. & Cowtan, K. D. (2020). *Acta Cryst.* D**76**, 713–723. 10.1107/S2059798320009080 PMC7397494 32744253

[bb13] Campbell, A. & Skoog, G. (2004). *J. Coll. Sci. Teach.* **33**, 24–26.

[bb14] Campbell, L. G., Mehtani, S., Dozier, M. E. & Rinehart, J. (2013). *PLoS One*, **8**, e79147. 10.1371/journal.pone.0079147 PMC3813606 24205372

[bb15] Cho, S.-Y. (2017). *SSRN*. https://doi.org/10.2139/ssrn.2902717.

[bb16] Cooper, J. & Weaver, K. D. (2003). *Gender and Computers: Understanding the Digital Divide.* Mahwah: Lawrence Erlbaum Associates.

[bb17] Costa, R., Serrano, M. A. & Salvador, A. (2016). *Psicothema*, **28**, 66–70. 10.7334/psicothema2015.166 26820426

[bb18] Cowtan, K., Rohde, R. & Hausfather, Z. (2018). *Q. J. R. Meteorol. Soc.* **144**, 670–681.

[bb19] Cowtan, K. & Way, R. G. (2014). *Q. J. R. Meteorol. Soc.* **140**, 1935–1944.

[bb20] Easterly, D. M. & Ricard, C. S. (2011). *J. Res. Adm.* **42**, 61–73.

[bb21] Eisenegger, C., Haushofer, J. & Fehr, E. (2011). *Trends Cogn. Sci.* **15**, 263–271. 10.1016/j.tics.2011.04.008 21616702

[bb22] Etzkowitz, H., Kemelgor, C. & Uzzi, B. (2000). *Athena Unbound: The Advancement of Women in Science and Technology.* Cambridge University Press.

[bb23] Fang, F. C. & Casadevall, A. (2015). *Infect. Immun.* **83**, 1229–1233. 10.1128/IAI.02939-14 PMC4363426 25605760

[bb24] Hansen, J., Ruedy, R., Sato, M. & Lo, K. (2010). *Rev. Geophys.* **48**, RG4004.

[bb25] Hausfather, Z., Cowtan, K., Clarke, D. C., Jacobs, P., Richardson, M. & Rohde, R. (2017). *Sci. Adv.* **3**, e1601207. 10.1126/sciadv.1601207 PMC5216687 28070556

[bb26] Hofstra, B., Kulkarni, V. V., Munoz-Najar Galvez, S., He, B., Jurafsky, D. & McFarland, D. A. (2020). *Proc. Natl Acad. Sci. USA*, **117**, 9284–9291. 10.1073/pnas.1915378117 PMC7196824 32291335

[bb27] Inckle, K. (2018). *Disabil. Soc.* **33**, 1372–1376.

[bb28] Inoue, Y., Takahashi, T., Burriss, R. P., Arai, S., Hasegawa, T., Yamagishi, T. & Kiyonari, T. (2017). *Sci. Rep.* **7**, 5335. 10.1038/s41598-017-05603-7 PMC5509644 28706184

[bb29] Kagan, R. L. (1979). *Averaging of Meteorological Fields.* St Petersburg: Gidrometeoizdat.

[bb30] Kahneman, D. (2011). *Thinking, Fast and Slow.* New York: Macmillan.

[bb31] Lincoln, A. E., Pincus, S., Koster, J. B. & Leboy, P. S. (2012). *Soc. Stud. Sci.* **42**, 307–320. 10.1177/0306312711435830 22849001

[bb32] Maccoby, E. E. (1990). *Am. Psychol.* **45**, 513–520. 10.1037//0003-066x.45.4.513 2186679

[bb33] Miller, B. (2013). *Synthese*, **190**, 1293–1316.

[bb34] Neumann, C. J. (2007). *EMBO Rep.* **8**, 202–206. 10.1038/sj.embor.7400913 PMC1808036 17330061

[bb35] Oreskes, N. (2019). *Why Trust Science?* Princeton University Press.

[bb36] Pauling, L. & Corey, R. B. (1953). *Proc. Natl Acad. Sci. USA*, **39**, 84–97. 10.1073/pnas.39.2.84 PMC1063734 16578429

[bb37] Pell, A. N. (1996). *J. Anim. Sci.* **74**, 2843–2848. 10.2527/1996.74112843x 8923199

[bb38] Pol, H. E. H., Cohen-Kettenis, P. T., Van Haren, N. E., Peper, J. S., Brans, R. G., Cahn, W., Schnack, H. G., Gooren, L. J. & Kahn, R. S. (2006). *Eur. J Endocrinol.* **155**, S107–S114.

[bb39] Powell, K. (2018). *Nature*, **558**, 19–22. 10.1038/d41586-018-05316-5 29875493

[bb40] Roper, R. L. (2019). *Microbiol. Mol. Biol. Rev.* **83**, e00018-19. 10.1128/MMBR.00018-19 PMC6710458 31315903

[bb41] Shansky, R. M. (2019). *Science*, **364**, 825–826. 10.1126/science.aaw7570 31147505

[bb42] Shen, H. (2013). *Nature*, **495**, 22–24. 10.1038/495022a 23467149

[bb43] Tannen, D. (2010). *Sci. Am. Mind*, **21**, 54–59.

[bb44] Tsai, W. (2002). *Organ. Sci.* **13**, 179–190.

[bb45] Wood, W. & Eagly, A. H. (2012). *Adv. Exp. Soc. Pyschol.* **46**, 55–123.

